# Pilot Scheme of Health Policy in Stroke Adjuvant Acupuncture Therapy for Acute and Subacute Ischemic Stroke in Taiwan

**DOI:** 10.1155/2011/689813

**Published:** 2011-04-18

**Authors:** Yi-Chia Wei, Mao-Feng Sun, Ku-Chou Chang, Chee-Jen Chang, Yu-Chiang Hung, Yu-Jr Lin, Hsien-Hsueh Elley Chiu

**Affiliations:** ^1^Department of Neurology, Chang Gung Memorial Hospital at Keelung, Keelung 20401, Taiwan; ^2^Department of Acupuncture, China Medical University Hospital, Taichung 40447, Taiwan; ^3^School of Chinese Medicine, China Medical University, Taichung 40402, Taiwan; ^4^Department of Neurology, Division of Cerebrovascular Diseases, Chang Gung Memorial Hospital—Kaohsiung Medical Center, Kaohsiung 83301, Taiwan; ^5^Center of Discharge Planning Service, Chang Gung Memorial Hospital—Kaohsiung Medical Center, Kaohsiung 83301, Taiwan; ^6^College of Medicine, Chang Gung University, Kaohsiung 83301, Taiwan; ^7^Yuh-Ing Junior College of Health Care and Management, Kaohsiung 80776, Taiwan; ^8^Graduate Institute of Clinical Medical Sciences, Chang Gung University, Taoyuan 33302, Taiwan; ^9^Clinical Informatics and Medical Statistics Research Center, Chang Gung University, Taoyuan 33302, Taiwan; ^10^Department of Traditional Chinese Medicine, Division of Internal Medicine, Chang Gung Memorial Hospital—Kaohsiung Medical Center, Kaohsiung 83301, Taiwan; ^11^Resource Center for Clinical Research, Chang Gung Memorial Hospital at Linkou, Taoyuan 33305, Taiwan; ^12^Department of Traditional Chinese Medicine, Division of Acupuncture and Chinese Traumatology, Chang Gung Memorial Hospital—Kaohsiung Medical Center, Kaohsiung 83301, Taiwan

## Abstract

To reduce the health care burden of strokes, the Taiwan Department of Health launched the Pilot Scheme of the Health Policy in Stroke Adjuvant Acupuncture Therapy (HPSAAT) in 2006. This cross-sectional, hospital-based, match-controlled study at Chang Gung Memorial Hospital-Kaohsiung Medical Center during 2006*∼*2008 retrospectively evaluated the clinical characteristics of acute and subacute ischemic stroke patients who electively joined the HPSAAT. The study also evaluated the safety and clinical benefits of adjuvant acupuncture in treating acute and subacute ischemic stroke patients. Twenty-six HPSAAT participants and 52 age-sex matched random controls were enrolled. The stroke baseline of the HPSAAT participants was more severe than the non-HPSAAT controls. Although the stroke severity closely correlates to mortality and comorbidity, this study noted no significant complications in the HPSAAT participants during the acupuncture treatment course. Adjuvant acupuncture was considered safe at the acute and subacute stages of ischemic stroke. Due to uneven baseline severity, the clinical benefits in reducing neurological deficits and functional recovery were not concluded in this study.

## 1. Introduction

As one of the leading causes of death, strokes contribute to a worldwide health care burden [1, 2]. Acupuncture has long been applied in treating stroke patients and has thus been recommended by the World Health Organization [3]. The National Health Insurance (NHI) is the social insurance that has covered the medical expenses of over 99% of 23.0 million Taiwanese citizens since 1995 [4–6]. In Taiwan, acupuncture prevailed in one fifth of NHI beneficiaries [7]. Aiming to improve the health care of stroke patients, the Department of Health launched an NHI-sponsored national project in 2006, namely the Pilot Scheme of the Health Policy in Stroke Adjuvant Acupuncture Therapy (HPSAAT). The HPSAAT promotes integration of traditional Chinese medicine (TCM) in conventional stroke care. In 2010, the HPSAAT has been conducting in 27 medical centers and local hospitals under an annual budget of 2.7 million US dollars. Patients with newly onset stroke (during the past year) receive support and elective acupuncture. Both inpatient treatment and outpatient clinic visits are covered.

Along the clinical course of ischemic stroke, the most important period of recovery is at the acute and subacute stage [8]. In this study, we sought to identify the characteristics of acute and subacute stroke patients who electively chose to participate in the HPSAAT. We also aimed to evaluate the safety of adjuvant acupuncture in stroke inpatient care.

Among patients with stroke, the major cause of death is from medical complications [9] that mostly develop within the first 6 weeks after stroke onset [10]. The incident rate of medical complications increases with the baseline severity of stroke. A higher score of the National Institution of Health Stroke Scale (NIHSS) [11, 12] or a higher dependency at stroke onset [10] is correlated to an increase of medical complications. Among all medical complications during acute and subacute stroke, infection is the most common medical complication [13] and is an independent predictor of mortality and poor functional outcome [9, 14, 15]. The high prevalence of poststroke infections, as well as other postCNS (central nervous system) injury infections, has been supposed to be the consequence of CNS immunodepression with imbalanced subsets of helper T cells [16, 17]. In acupuncture theory, correcting imbalances is the ultimate goal of needling. Both proinflammatory and inflammatory effects have been observed in acupuncture studies [18]. The postacupuncture sequential change of peripheral blood leukocyte subpopulation and cytokines reveals immune-boosting effects [19, 20]. Based on the previous findings, we hypothesize that acupuncture has the potential to correct CNS injury-induced immunodepression and reduce the infectious complications of stroke patients. In this study, we attempted to use age-sex matched control to evaluate the clinical benefits of adjuvant acupuncture in reducing medical complications in HPSAAT participants.

The other major burden in stroke care involves neurological deficits and functional impairment. The acute and subacute stages account for the golden time of stroke recovery [8]. The plasticity of the brain significantly affects stroke recovery [21]. In recent systematic reviews of acupuncture in acute ischemic stroke, potential therapeutic effects have been noticed [22, 23]. Acupuncture increases regional cerebral blood flow in the hypoperfusion area surrounding the ischemic core and in the sensorimotor area of the affected and unaffected hemisphere of the brain in ischemic stroke patients [24]. A functional MRI study confirmed that acupuncture elicits higher signals in the somatosensory area of poststroke brains [25]. In this study, we compared the neurological improvement between the HPSAAT participants and age-sex matched controls.

## 2. Method

### 2.1. Study Design and Patients

This research involved a cross-sectional, hospital-based, matched-control study conducted at Chang Gung Memorial Hospital-Kaohsiung Medical Center (KCGMH). The study was approved by the institutional review board of KCGMH. Acute and subacute stroke patients admitted to the neurology ward and neurology intensive care unit (NICU) from 1 July 2006 to 30 June 2008 were assessed for eligibility. Ischemic stroke is defined by ICD-9 code 434. Only admissions for initial management of acute and subacute ischemic stroke were included. Re-admissions of a single patient during the investigation period were excluded. Among ischemic stroke patients, some patients electively joined the HPSAAT to receive adjuvant acupuncture. Occasionally, families of patients with cognitive impairment made the decision regarding HPSAAT participation. After patients began participating in the HPSAAT, TCM doctors received a consultation sheet, visited the HPSAAT participants, and began the treatment course. 

**Figure 1:**
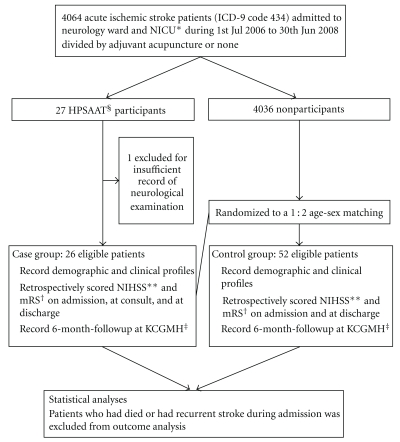
Participant flowsheet. *NICU, neurology intensive care unit. **NIHSS, National Institutes of Health Stroke Scale. Scored by one independent physician with certification of American Heart Association Professional Education Center website. ^†^mRS, modified Rankin Scale. ^‡^KCGMH, the Chang Gung Memorial Hospital-Kaohsiung Medical Center. ^§^HPSAAT, the Pilot Scheme of the Health Policy in Stroke Adjuvant Acupuncture Therapy. HPSAAT provides stroke patients elective acupuncture at outpatients and inpatients departments during the first year after stroke onset.

 In this study, HPSAAT participants were allocated to case groups. Matched control group members were randomly selected among the nonHPSAAT participants by 1 : 2 age-sex matching [26]. Those cases whose medical records did not present sufficient neurological descriptions for NIHSS scoring were excluded. The enrolled cases were retrieved for full chart review of the admission course and 6-month period of poststroke followup at KCGMH (Figure 1).

### 2.2. Procedures

All patients received concomitant stroke care in the neurology ward and/or in NICU. In addition, experienced TCM doctors visited the HPSAAT participants three times per week and performed acupuncture treatment. The acupuncture point selection was determined by doctor clinical evaluation in accordance with TCM principles. After manual acupuncture, the disposable stainless needles were retained for 15 minutes. When clinical conditions deteriorated or infectious conditions occurred, doctors of both neurology and TCM could evaluate the patients and decide if acupuncture should be administered.

### 2.3. Data Collection

The demographic profiles recorded age, sex, and body mass index. The clinical profile regarding strokes recorded stroke types, lesion sites, previous strokes, and risk factors. The population profile recorded patient sources, dispositions, and timescale of treatment. The medical resource profile recorded thrombolytic therapy (r-tPA), NICU stay, craniectomy or shunt implantation, mechanical ventilation, bedside physical therapy, and length of stay. Confounding factors of outcome were assessed by a baseline NIHSS [27] and a baseline modified Rankin Scale (mRS) [28]. We retrospectively scored the NIHSS [29, 30] and mRS by an independent clinical investigator certified by the American Heart Association professional education center [31].

Neurological complications were recorded as recurrent stroke during admission and stroke-in-evolution [32]. Medical complications during admission were evaluated by urinary tract infection, pneumonia, and cellulitis [13]. Also the stress events represented by gastrointestinal (GI) bleeding episodes, hemodynamic stability evaluated by vital sign at 09:00 a.m. of the day next to admission day and of the day before discharge day. Significant adverse events of acupuncture were reviewed [33]. We also traced patient admission records and outpatient visits at KCGMH for a 6-month period. Patients who had died or had a recurrent stroke during admission were separated from outcome analysis.

### 2.4. Statistical Analysis

Categorical data were expressed by frequency (percentage) and were examined by a chi-squared test. Continuous data were expressed by mean (standard deviation) and were examined by an independent *t*-test. A *P-*value <  .05 in a 2-tailed test was considered to be statistically significant. Data were analyzed using a commercial software program, SPSS version 17.0 for Windows; SPSS Inc.

## 3. Results

This study involved 273 HPSAAT participants, most of them were in the stroke recovery stage and admitted in rehabilitation wards. Only 27 participants with acute and subacute ischemic stroke were eligible for the case group. One case was further excluded due to insufficient neurological examination records for retrospective NIHSS scoring, leaving 26 participants in the case group. Fifty-two age-sex matched controls were randomly selected from 4,036 acute and subacute ischemic stroke patients who did not participate in the HPSAAT but received conventional treatment indiscriminately (Figure 1).

The characteristic profile of the HPSAAT participants and age-sex matched nonHPSAAT controls showed no difference in demographics, stroke type (Table 1(a)), or patient source (Table 1(b)). However, the HPSAAT participants seemed to have longer treatment courses, including length of stay in NICU, in the neurology ward, and in total (32.9 ± 21.9 : 13.8 ± 16.1 days, *P* < .001). Compared to the nonHPSAAT controls, the HPSAAT participants tended to stay in health care institutions after discharge from the neurology ward and/or NICU, including the rehabilitation ward, TCM ward, and long-term care units (Table 1(b)). Bedside physical therapy was more prevalently applied to the HPSAAT participants (92.3% : 53.8%, *P* = .001; Table 1(c)).

The HPSAAT participants in the neurology ward and NICU started adjuvant acupuncture an average of 17.7 ± 14.4 days after stroke onset and continued the treatment course for 17.4 ± 16.2 days with 6.7 ± 6.4 acupuncture sessions (Table 1(b)). The safety of acupuncture was evaluated in a detailed chart review. No vital sign instability was noted among the HPSAAT participants during acupuncture. The baseline vital sign on admission was not significantly different between groups; whereas, the HPSAAT participants presented lower diastolic blood pressure (76.8 ± 8.5 : 82.1 ± 9.7 mmHg, *P* < .05), lower mean arterial pressure (95.6 ± 8.9 : 100.7 ± 10.7 mmHg, *P* < .05), and faster heart rate (79.1 ± 8.1 : 73.7 ± 7.2 beat per minute, *P* < .01) than the nonHPSAAT controls. The adverse event survey revealed no needle sickness. In one participant, left-arm cellulitis developed 8 days after acupuncture initiation (cause undetermined). An intravenous antibiotic was administered and the acupuncture treatment was uninterrupted and completed until the patient was discharged home. Regarding another participant who had begun acupuncture treatment in the neurology ward, the acupuncture treatment was interrupted after two sessions (7 days) of treatment because of sudden collapse and admission to NICU. The other 24 HPSAAT participants completed the acupuncture treatment course smoothly during the stay in the neurology ward and/or NICU.

**Figure 2:**
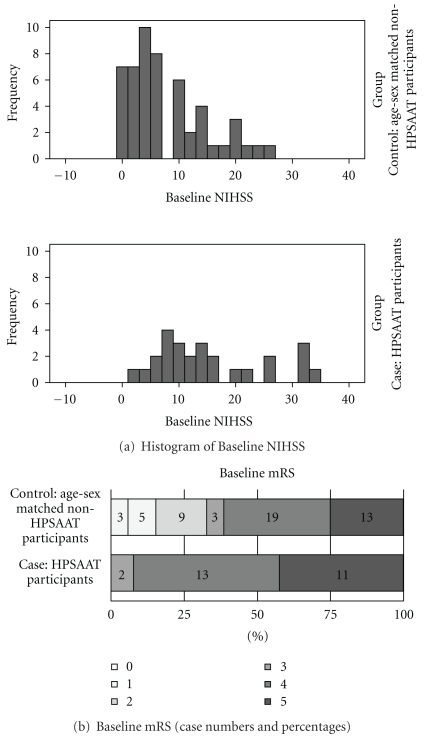
Baseline discrepancies between HPSAAT participants and age-sex matched nonparticipants. The histogram of baseline NIHSS showed the different distribution of severity between groups. There were more moderate to severe strokes among the HPSAAT participants with acute and subacute ischemic stroke than the age-sex matched nonHPSAAT controls (a). The number and percentage of baseline mRS showed that the HPSAAT participants were more dependent than the random controls (b).

The mortality and comorbidity analysis began from a baseline comparison. The baseline NIHSS was higher on average (14.9 ± 9.4 : 7.5 ± 7.0, *P* < .001; Table 2(a)) and was skewed more to the left in distribution (Figure 2(a)) among the HPSAAT participants than that of the nonHPSAAT controls. The baseline mRS of the HPSAAT participants was also more severe than that of the nonHPSAAT controls (dependent as mRS > 3 in 92.3% : 61.5%, *P* < .01), but was distributed similarly (Figure 2(b)). Three enrolled cases expired during admission. All mortalities occurred in the control group and were caused by severe infection with septic shock. A comparison of neurological and medical complications discovered more stroke-in-evolution (34.6% : 3.8%, *P* = .001) and urinary tract infection (46.2% : 23.1%, *P* < .05) in the HPSAAT participants than in the nonHPSAAT controls during stays in the neurology ward and/or NICU. However, more urinary tract infections occurred before initiating acupuncture (Table 2(b)). No difference was noted in the occurrences of recurrent stroke, pneumonia, cellulitis, or GI bleeding.

In the 6-month followup period, the HPSAAT participants tended to maintain regular outpatient visits to the rehabilitation department (20.0% : 2.1%, *P* < .05) and acupuncture department (32.0% : 0.0%, *P* < .001). Approximately seventy percent of cases in both groups continued following up in the neurology department for at least 6 months after stroke onset.

## 4. Discussion

In this study, the major finding, but also the major limitation, was the uneven baseline severity between the HPSAAT participants and the age-sex matched nonHPSAAT controls. The acute and subacute ischemic stroke patient with severe neurological impairment and significantly functional limitations was prone to join the HPSAAT for adjuvant acupuncture in addition to conventional treatment. This finding indicated the desire of major ischemic stroke patients and their families to pursue satisfying treatment modalities or a combination of treatment modalities. This finding also indicated that the developmental principle of acupuncture in treating ischemic stroke should be emphasized on moderate to severe patients.

However, the baseline discrepancy limited the effective comparison of neurological and functional outcomes. The alternative solution may come from data mining in the Chang Gung Stroke Registry Database, which includes clinical data of all stroke patients admitted to the four branches of Chang Gung Memorial Hospital since 2008. Searching by the NIHSS on admission could find the baseline equaled populations in the database.

The varied duration of stroke onset to acupuncture treatment (17.7 ± 14.4 days) and varied duration of acupuncture treatment (17.4 ± 16.2 days) among the HPSAAT participants also limited the comparison of benefits from adjuvant acupuncture in ischemic stroke. For more accurate generalization and more effective comparison, a randomized control trial is required.

Since the 1990s, the efficacy and effectiveness of acupuncture for acute and subacute stroke has been evaluated by many research groups. Although some study designs have been limited by the strength of evidence, systemic reviews revealed the potential benefits of acupuncture to patients with acute and subacute strokes [22, 23]. The HPSAAT is the first public health policy-modifying project to include acupuncture in stroke care. Previous evidence has suggested that the worse the baseline is, the worse the outcomes are. Moderate to severe strokes had poorer outcomes [34, 35], greater demand of long-term care [36, 37], and higher expense of medical costs [38]. The acute and subacute stages constitute the golden recovery period for ischemic stroke. From this study, we found that major ischemic stroke patients tended to choose adjuvant acupuncture in the acute and subacute stages. Future studies must clarify whether or not the HPSAAT reduced the financial burden. Moreover, the baseline severity also correlated to the increased poststroke infections [12]. In this study, a higher rate of urinary tract infection of the HPSAAT participants, but not of pneumonia or GI bleeding, was noted. The infections that occurred after starting acupuncture treatment did not seem significantly increased. Therefore, in addition to improving neurological impairment or functional recovery, acupuncture may play a role in eliminating medical complications of stroke. The immune-boosting potentials of acupuncture in modulating CNS injury-induced immunodepression of acute and subacute stroke is still worthy of further investigation.

The 6-month followup period revealed favorable compliance of the HPSAAT participants to continue acupuncture treatment after discharge. The needs of long-term functional recovery and the selection of acupuncture as a treatment modality was noticed in one third of the HPSAAT participants.

## 5. Conclusion

This cross-sectional study analyzed data from a single medical center to depict a brief summary of the HPSAAT in Taiwan. Adjuvant acupuncture care is a safe modality in the acute and subacute stages of ischemic stroke. Ischemic stroke patients with a severe baseline tended to participate in the HPSAAT. To validate the clinical benefits of adjuvant acupuncture care in acute and subacute ischemic stroke, a baseline equaled randomized control trial is warranted.

##  Statement of Contribution

We state the contribution of Yi-Chia Wei MD in data acquirement, data analysis, and manuscript drafting, Mao-Feng Sun MD/PhD in HPSAAT project supervision, Ku-Chou Chang MD in data acquiring and study supervision, Chee-Jen Chang PhD in study design, Yu-Chiang Hong MD/PhD in supervision of this study, Yu-Jr Lin MSc in performing statistical analysis, and Hsien-Hsueh Elley Chiu MD in conducting HPSAAT, conceiving and design the research, analyzing and interpreting the data, and making critical revision of the manuscript.

##  Conflicts of Interest 

All authors and contributors declared that no conflict of interest exists.

##  Funding

This study was supported by the Chung Gung Research Project, CMRPG880551. The funding source had no role in the study design, data collection, data analysis, data interpretation, or writing of the report. The corresponding author and the first author had full access to all data in the study and had final responsibility for the decision to submit the paper for publication.

## Figures and Tables

**Table 1:** Clinical characteristics.

	HPSAAT participants (*n* = 26)	Matched control : non HPSAAT (*n* = 52)	*P*-value
(a) Demographics			

Age, years*	70.2 (11.3)	70.2 (11.1)	Matched
Sex, male**	16 (61.5)	32 (61.5)	Matched
Body mass index, kg/m^2∗^	23.5 (3.2)	23.8 (3.6)	
Stroke ever**	14 (53.8)	26 (50.0)	
Stroke type**			
Ischemic	23 (88.5)	50 (96.2)	
Ischemic with hemorrhagic transformation	3 (11.1)	2 (3.7)	
Stroke lesion site**			
Right cerebral hemisphere	5 (19.2)	16 (30.8)	
Left cerebral hemisphere	8 (30.8)	15 (28.8)	
Cerebellum	0 (0.0)	2 (3.8)	
Brain stem	5 (19.2)	11 (21.2)	
Multiple infarcts (≥2 of above)	8 (30.8)	8 (15.4)	
Risk factors of stroke**			
Hypertension	20 (76.9)	33 (63.5)	
Diabetes mellitus	14 (53.8)	25 (48.1)	
Hypertriglyceridemia	7 (26.9)	10 (19.2)	
Hypercholesterolemia	11 (42.3)	17 (32.7)	
Atrial fibrillation	5 (19.2)	6 (11.5)	
Coronary artery disease	5 (19.2)	4 (7.7)	
Congestive heart failure	0 (0.0)	1 (1.9)	
Cigarette smoking	6 (23.1)	16 (30.8)	
Obesity	7 (31.8)	14 (33.3)	

(b) Patients' source, treatment course, and disposition			

Patient source**			
Emergency room	20 (76.9)	39 (75.0)	
Referred from other hospital	3 (11.5)	4 (7.7)	
Outpatients	1 (3.8)	9 (17.3)	
Referred from other ward in hospital	2 (7.7)	0 (0.0)	
Timescale of treatment, days*			
Onset to ward	2.9 (4.7)	2.3 (3.1)	
Onset to acupuncture	17.7 (14.4)	—	
Duration of acupuncture^†^	17.4 (16.2)	—	
Acupuncture session, times	6.7 (6.4)	—	
Interval of acupuncture sessions	2.9 (1.5)	—	
Length of stay, days^∗,‡^			
In NICU	11.6 (14.1)	2.2 (4.7)	<.01
In neurology ward	19.2 (8.6)	11.6 (12.7)	<.01
In RCC	2.0 (5.5)	0.0 (0.0)	
Total	32.9 (21.9)	13.8 (16.1)	<.001
Disposition^∗∗,‡^			
Home	16 (64.0)	46 (95.8)	.001
Rehabilitation ward	4 (16.0)	0 (0.0)	<.05
Traditional Chinese medicine ward	1 (4.0)	0 (0.0)	
Long-term care unit^§^	5 (20.0)	2 (4.2)	<.05

(c) Medical resource use**			

Received r-tPA	0 (0.0)	0 (0.0)	
Ever NICU stay	17 (65.4)	15 (28.8)	<.01
Craniectomy or shunt implantation	2 (7.7)	0 (0.0)	
Mechanical ventilation	4 (15.4)	2 (3.8)	
Received bedside physical therapy	24 (92.3)	28 (53.8)	.001

*Data are given as mean (SD). **Data are given as number (percentage). ^†^Mortality was excluded (case = 0, control = 3). ^‡^Mortality (case = 0, control = 3) and patients with recurrent stroke during admission (case = 1, control = 1) were excluded. ^§^Long-term care unit included local hospital and nursing home. Abbreviation: HPSAAT: the Pilot Scheme of the Health Policy in Stroke Adjuvant Acupuncture Therapy. NICU: neurology intensive care unit. RCC: respiratory care center. r-tPA: thrombolytic therapy.

**Table 2:** Mortality and comorbidity during admission and in six-month-followup.

	HPSAAT participants (*n* = 26)	Matched control : non HPSAAT (*n* = 52)	*P*-value
(a) Baseline comparison: factors affecting mortality and comorbidity			

Neurological impairment*			
Baseline NIHSS scores	14.9 (9.4)	7.5 (7.0)	<.001
Interquartile range	7–22	2-11.75	
NIHSS at consult	16.6 (8.8)	—	
NIHSS at discharge^‡^	15.4 (8.3)	5.6 (5.5)	<.001
Functional impairment**			
Baseline mRS > 3	24 (92.3)	32 (61.5)	<.01
mRS > 3 at consult	25 (96.2)	—	
mRS > 3 at discharge^‡^	22 (88.0)	24 (50.0)	.001

(b) Mortality and comorbidity during admission**			

Mortality during admission	0 (0.0)	3 (5.8)	
Neurological complications			
Recurrent stroke	1 (3.8)	1 (1.9)	
Stroke in-evolution after admission	9 (34.6)	2 (3.8)	.001
Medical complications			
Urinary tract infection (total)	12 (46.2)	12 (23.1)	<.05
Before acupuncture	7 (26.9)	—	
After acupuncture	5 (19.2)	—	
Pneumonia (total)	9 (34.6)	11 (21.2)	
Before acupuncture	9 (34.6)	—	
After acupuncture	0 (0.0)	—	
Cellulitis (total)	2 (7.7)	1 (1.9)	
Before acupuncture	1 (3.8)	—	
After acupuncture	1 (3.8)	—	
Gastrointestinal bleeding	4 (15.4)	8 (15.4)	
Before acupuncture	4 (15.4)	—	
After acupuncture	0 (0.0)	—	

(c) Six-month followup in KCGMH^∗∗,‡^			

Ever readmission due to acute disorders	2 (8.0)	7 (14.6)	
Recurrent stroke within 6 months	0 (0.0)	1 (2.1)	
Expire within 6 months	0 (0.0)	2 (4.2)	
Outpatient followup^∗∗,‡^			
In neurology department			
First 3 months	19 (76.0)	37 (77.1)	
6 months	17 (68.0)	37 (77.1)	
In rehabilitation department			
First 3 months	5 (20.0)	2 (4.2)	<.05
6 months	5 (20.0)	1 (2.1)	<.05
In acupuncture department			
First 3 months	9 (36.0)	0 (0.0)	<.001
6 months	8 (32.0)	0 (0.0)	<.001

*Data are given as mean (SD). **Data are given as number (percentage). ^†^Mortality was excluded (case = 0, control = 3). ^‡^Mortality (case = 0, control = 3) and patients with recurrent stroke (case = 1, control = 1) during admission were excluded. Abbreviation: HPSAAT, the Pilot Scheme of the Health Policy in Stroke Adjuvant Acupuncture Therapy. NIHSS: National Institutes of Health Stroke Scale. mRS: modified Rankin Scale. mRS > 3 was defined as dependent status. KCGMH: the Chang Gung Memorial Hospital—Kaohsiung Medical Center.
